# Impairment of autophagy in the central nervous system during lipopolysaccharide-induced inflammatory stress in mice

**DOI:** 10.1186/s13041-014-0056-z

**Published:** 2014-08-27

**Authors:** Arnaud François, Faraj Terro, Nathalie Quellard, Béatrice Fernandez, Damien Chassaing, Thierry Janet, Agnès Rioux Bilan, Marc Paccalin, Guylène Page

**Affiliations:** 1 EA3808 molecular Targets and Therapeutic of Alzheimer’s disease, University of Poitiers, Poitiers F-86073, France; 2 Laboratory of Histology and Biology, Faculty of Medicine, University of Limoges, Limoges F-87025, France; 3 Service d’histologie et de cytogénétique, Hôpital de la Mère et de l’Enfant, Limoges F-87000, France; 4 Pathology Department, Poitiers University Hospital, Poitiers F-86021, France; 5 Geriatrics Department, Poitiers University Hospital, Poitiers F-86021, France; 6 CMRR, Poitiers University Hospital, Poitiers F-86021, France; 7 CIC-P 1402, Poitiers University Hospital, Poitiers F-86021, France; 8 Pôle Biologie Santé, Université de Poitiers, Bâtiment B36/B37 Secteur β - Niveau 0, 1 Rue Georges Bonnet, Poitiers 86073 Cedex 9, France

**Keywords:** Inflammation, Autophagy, IL-1β, Brain, Mouse, Lipopolysaccharide

## Abstract

**Background:**

Current evidence suggests a central role for autophagy in many neurodegenerative diseases including Alzheimer’s disease, Huntington’s disease, Parkinson’s disease and amyotrophic lateral sclerosis. Furthermore, it is well admitted that inflammation contributes to the progression of these diseases. Interestingly, crosstalks between autophagy and inflammation have been reported in vitro and at the peripheral level such as in Crohn’s disease. However, the impact of systemic inflammation on autophagic components in the brain remains to be documented. Therefore, this study monitored autophagy markers after acute and chronic lipopolysaccharide (LPS)-induced inflammatory stress in mice.

**Results:**

We showed that acute inflammation, 24 h post-intraperitoneal 10 mg/kg LPS, substantially increased cytokine production (Interleukin(IL)-1β, Tumor necrosis factor (TNF)-α and IL-6), decreased the levels of autophagy markers (Beclin-1, p62 and LC3 II) and reduced p70S6K activation in cortex and hippocampus. In hippocampus, IL-1β levels and LC3 II expression were positively and highly correlated and a negative correlation was noted between TNF-α levels and p70S6K activation. Chronic inflammation by injection of 0.5 mg/kg LPS every three days during three months led to a moderate IL-1β production and decreased TNF-α levels. Interestingly, Beclin-1 and LC3 II levels decreased while those of p62 increased. Cortical IL-1β levels positively correlated with Beclin-1 and LC3 II and on the contrary inversely correlated with p62.

**Conclusion:**

The present study is the first showing links between IL-1β-mediated inflammation and autophagy in the brain. It could open to new therapeutic strategies in brain diseases where regulation impairment of inflammation and autophagy progress with the severity of diseases.

## Background

Autophagy is a major catabolic pathway in eukaryotic cells and is responsible for the degradation in the lysosome of long-lived proteins and altered or unwanted organelles. Autophagy not only plays a crucial role in the maintaining of cell homeostasis and protein quality control but also constitutes an adaptive response to nutritional stress and protects the cells against microbial and viral pathogens and damaged structures. The molecular mechanisms controlling autophagy processes are complex. The mammalian target of rapamycin (mTOR) is an S/T kinase playing a central role in the control of autophagy [1]–[5]. However autophagy can be induced in mTOR-independent manners, in that cases it involves inositol, calcium, 5’-adenosine monophosphate-activated protein kinase (AMPK), Jun kinase (JNK)-beclin-1 complex and Sirtiun-1 [6]–[11]. Through this regulation, autophagy controls cell metabolism, apoptosis, protein secretion and cell-mediated immune responses [12]–[16]. The role of autophagy in inflammatory diseases was initially established through genome-wide association studies (GWAS) showing that polymorphisms in autophagy-associated genes, such as ATG16L1 and IRGM, are linked to Crohn’s disease, the well-known inflammatory bowel disease [17]–[20]. In addition, polymorphisms in autophagy-associated genes have been associated with other inflammatory diseases such as systemic lupus erythematosus [21], asthma [22] and rheumatoid arthritis [23]. Besides, autophagy has been recognized to have an anti-inflammatory action since the production of interleukine (IL)-1β and IL-18 was increased in the absence of functional ATG16L1 (a key protein of the ubiquitin-like conjugation system Atg5-Atg12 ~ Atg16) in a mouse model of Crohn’s disease [24]. Several convergent reports showed that autophagy interferes with inflammasome (complex involved in IL-1β maturation) activation by targeting ubiquitinated aggregates of inflammasome components for destruction [25]–[28]. Conversely, altered proteostasis has been shown recently to activate inflammasome [29]. In addition, NF-κB signalling pathway has been demonstrated to be involved in the induction of autophagy [30],[31] and the pro-inflammatory cytokines IL-1β, TNF-α and IFN-γ were shown to activate autophagy under infectious stimuli [32],[33] contrary to IL-4 and IL-13 [34].

The interconnections between autophagy and inflammation were mainly described at peripheral level in particular in inflammatory bowel diseases [35], type 2 diabete [36], cardiac disorders [37], cystic fibrosis [38]. However, such interconnections remain to be investigated in the central nervous system (CNS) as autophagy alterations and inflammation are also two components of neurodegenerative disorders such as Parkinson’s disease (PD), Alzheimer’s disease (AD), Huntington’s disease (HD), Amyotrophic Lateral Sclerocis (ALS) [39].

Here, we investigated the impact of an inflammatory reaction on the autophagic process in the CNS in vivo. Neuroinflammation was triggered by intraperitoneal lipopolysaccharide (LPS) injection in mice as previously described in rodents [40]–[44]. We showed that according to the inflammatory stress severity (acute versus chronic), neuroinflammation differently altered autophagy in the brain underlying a potential role of cortical IL-1β in the alterations of autophagy in the CNS. These findings reported for the first time relationships between inflammation and autophagy in CNS and could open to new therapeutic strategies in brain diseases where regulation impairment of inflammation and autophagy progress with the severity of diseases.

## Results

As indicated, we first studied the impact of a peripheral acute inflammatory stress induced by LPS on cerebral autophagy in wild-type B6C3F1 mice. Preliminary experiments were performed using 2 doses of LPS: either one i.p. injection at 1 mg/kg, and sacrifice 3 h after or 3 i.p. injections at 3 mg/kg per 24 h before sacrifice. However, no statistically significant production of IL-1β, TNF-α and IL-6 was measured compared to saline group mice (Tables 1 and 2). Consequently, we used a higher LPS dose of 10 mg/kg, 3 i.p injections per 24 h, in order to trigger relevant inflammatory response in our experimental settings. This relatively high dose has been previously reported, by other authors, to be lethal 48 h after LPS injection [45]. In our study, animals received 1, 2 or 3 i.p injections and were sacrificed 24 h after LPS injections. In these conditions, no mortality was observed.

**Table 1:** Cortical and hippocampus cytokine levels in mice treated with 1 mg/kg of LPS

	**Cortex**		**Hippocampus**	
	**NaCl**	**LPS**	**NaCl**	**LPS**
**IL-1β**	28.07 ± 7.53	35.05 ± 15.55	62.65 ± 16.35	40.4 ± 14.80
**TNF-α**	3.57 ± 1.30	2.83 ± 0.80	6.53 ± 1.42	1.47 ± 0.62
**IL-6**	3.06 ± 1.02	6.07 ± 1.16	3.23 ± 0.88	3.05 ± 0.96

Levels of IL-1β, TNF-α and IL-6 measured by ELISA in cortex and hippocampus of 3-months old mice treated with one i.p. injection of LPS at 1 mg/kg or saline (0.9% NaCl) and sacrificed after 3 hours. Cytokine levels are expressed in pg/mg of protein. Results are ± SEM of 3 mice per group. ND: not detectable, under the limit of detection.

**Table 2:** Cortical and hippocampus cytokine levels in mice treated with 3 mg/kg of LPS

	**Cortex**		**Hippocampus**	
	**NaCl**	**LPS**	**NaCl**	**LPS**
**IL-1β**	23.94 ± 6.71	18.05 ± 1.44	35.1 ± 1.22	49.32 ± 8.25
**TNF-α**	1.24 ± 0.29	1.78 ± 0.10	1.62 ± 0.20	1.67 ± 0.56
**IL-6**	3.83 ± 0.95	3.56 ± 0.24	5.33 ± 0.26	3.87 ± 0.08

Levels of IL-1β, TNF-α and IL-6 measured by ELISA in cortex and hippocampus of 3-months old mice treated with 3 i.p. injections of LPS at 3 mg/kg or saline (0.9% NaCl) per 24 h before sacrifice. Cytokine levels are expressed in pg/mg of protein. Results are ± SEM of 3 mice per group.

### Pro-inflammatory cytokine levels after acute LPS-induced inflammatory stress

In saline group mice, no statistically significant variations of cytokine levels were observed in cortex and hippocampus whatever the design of treatment (see Additional file 1: Table S1 and S2). Therefore, results for NaCl correspond to the mean in each figure.

**Figure 1:**
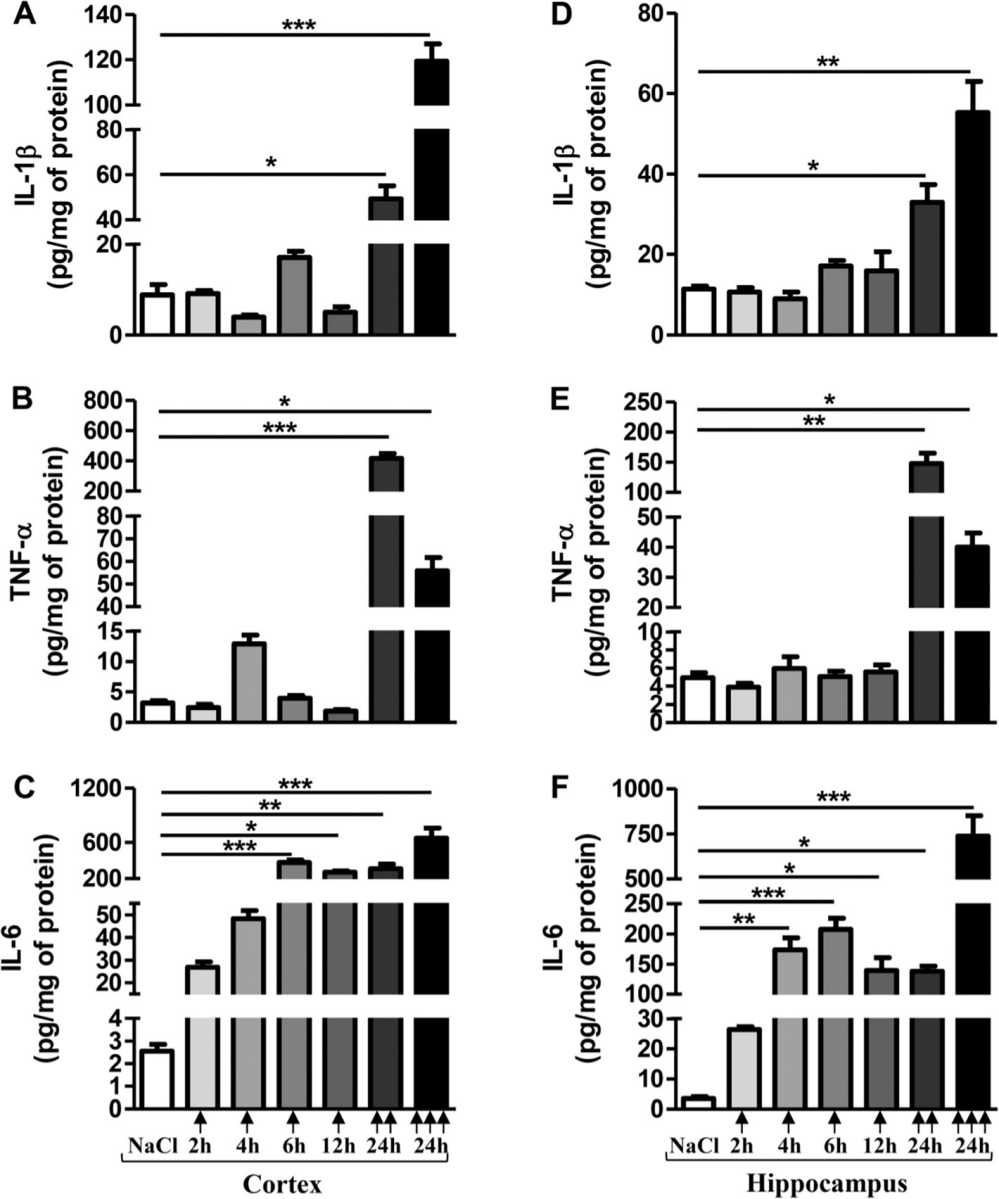
**Pro-inflammatory cytokine levels after acute LPS-induced inflammatory stress.** IL-1β, TNF-α and IL-6 levels in cortex (**A**, **B**, **C**, respectively) and hippocampus (**D**, **E**, **F**, respectively) of mice treated with a single () or two () or three () i.p. injections of LPS at 10 mg/kg or vehicle (0.9% NaCl) were measured by ELISA assay. Mice were sacrificed 2, 4, 6 or 12 h after a single injection or 24 h after two or three injections. Cytokine levels were expressed in pg/mg protein. Results are mean ± SEM for 6 mice in each group. *p < 0.05, **p < 0.01, ***p < 0.001 compared to 0.9% NaCl-injected mouse group by Kruskal-Wallis test with a Dunns multiple comparison test.

After 24 h, two or three injections of LPS at a dose of 10 mg/kg increased IL-1β, TNF-α and IL-6 levels in the cortex and in the hippocampus (Figure 1). Cortical IL-1β levels were 5.6- and 13.5-fold higher compared to the corresponding vehicle-injected mice with two and three injections, respectively (Figure 1A). Hippocampal IL-1β levels were 3 and 5 times higher (Figure 1D). Cortical TNF-α levels increased by 131 and 17.5 times with two or three injections, respectively. Hippocampal TNF-α levels were 30 and 8 times higher (Figure 1B and E). For IL-6, cortical levels were 121 or 254 times higher with two or three injections, respectively and hippocampal levels were 40 or 212 times higher, compared to those measured in vehicle-treated mice (Figure 1C and F). Unlike IL-1β and TNF-α, IL-6 levels were significantly increased in the cortex from 6 h (148-fold) and from 4 h in the hippocampus (50-fold).

### Changes in autophagic factors after acute LPS-induced inflammatory stress

**Figure 2:**
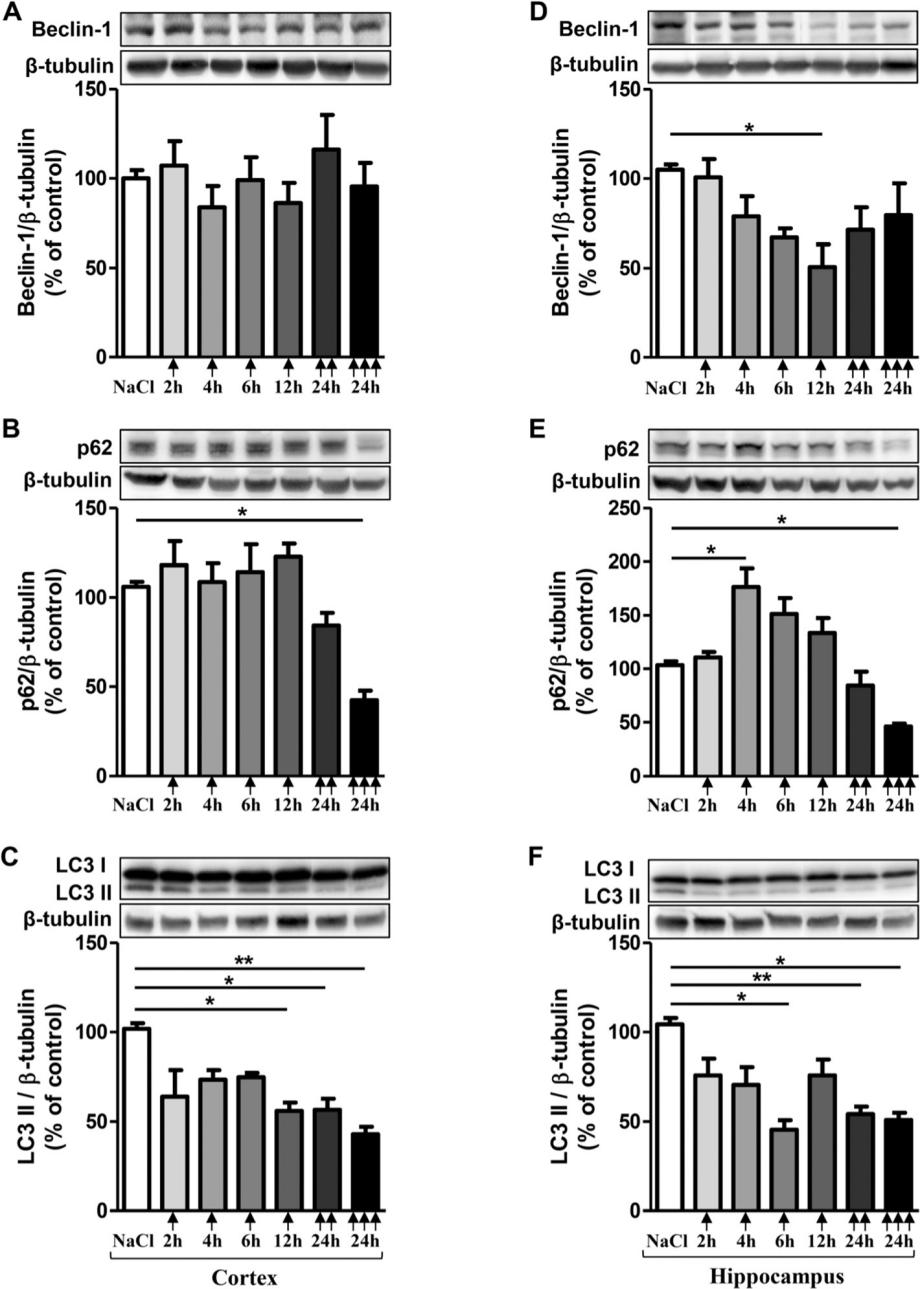
**Changes in autophagic factors after acute LPS-induced inflammatory stress.** Representative immunoblots showed the immunoreactivity of Beclin-1 **(A)**, p62 **(B)** LC3 II **(C)** in cortex and Beclin-1 **(D)**, p62 **(E)** LC3 II **(F)** in hippocampus from mice treated with a single () or two () or three () i.p. injections of LPS at 10 mg/kg or vehicle (0.9% NaCl). Mice were sacrificed 2, 4, 6 or 12 h after a single injection and 24 h after two and three injections. Semi-quantitative analysis of immunoblot was performed using Gene Tools software (Syngene, Ozyme France). The immunoreactivity of protein was normalized to β-tubulin immunoreactivity. The results are expressed as arbitrary units (% of 0.9% NaCl-injected mice group set at 100%). Results are mean ± SEM for 6 mice in each group. *p < 0.05, **p < 0.01 compared to 0.9% NaCl-injected mouse group by Kruskal-Wallis test with a Dunns multiple comparison test.

As for inflammatory markers, no statistically significant variations of autophagic markers were observed in cortex and in hippocampus whatever the design of treatment in saline-injected group mice (see Additional file 1: Table S3 and S4). Therefore, results for NaCl correspond to the mean in each panel of Figure 2.

To determine whether autophagy changes occured after a peripheral acute inflammatory stress, Beclin-1, p62, LC3 I and LC3 II were investigated. Beclin-1 is a key component of the class III PI3K (Phosphatidylinositide 3-kinases) complex which is involved in the initiation of autophagosome formation [46]; p62 is an autophagic receptor which recognizes ubiquitinylated proteins and interacts with LC3 II at the forming autophagosome [47]; LC3 is present in free cytoplasmic form as LC3 I which, when is conjugated to phosphatidylethanolamine (through an ubiquitin-like conjugation reaction) of the membrane of autophagosome, produces LC3 II form, a useful marker of autophagic vacuoles [47].

Beclin-1 expression was affected only in hippocampus with a decrease by 52% after 12 h post-injection (Figure 2A and D). A higher inflammatory stress with two or three LPS injections did not significantly change Beclin-1 expression (Figure 2D).

For p62, a single LPS injection induced an increase by 71% after 4 h and return to basal line after 6 and 12 h in the hippocampus (Figure 2E). No changes in p62 levels were observed in the cortex after a single injection (Figure 2B). On the contrary, a decrease in p62 levels of 18-20% with two injections and of 55-60% with three injections in both cortical and hippocampal area were observed (Figure 2B and E).

For LC3 marker, any change was observed for LC3 I both in cortex and hippocampus regardless the time post- LPS injection. However, LC3 II significantly decreased at 12 h in the cortex (45%), at 6 h in the hippocampus (56.5%) and at 24 h after two or three injections (45-58%) in both areas (Figure 2C and F).

**Figure 3:**
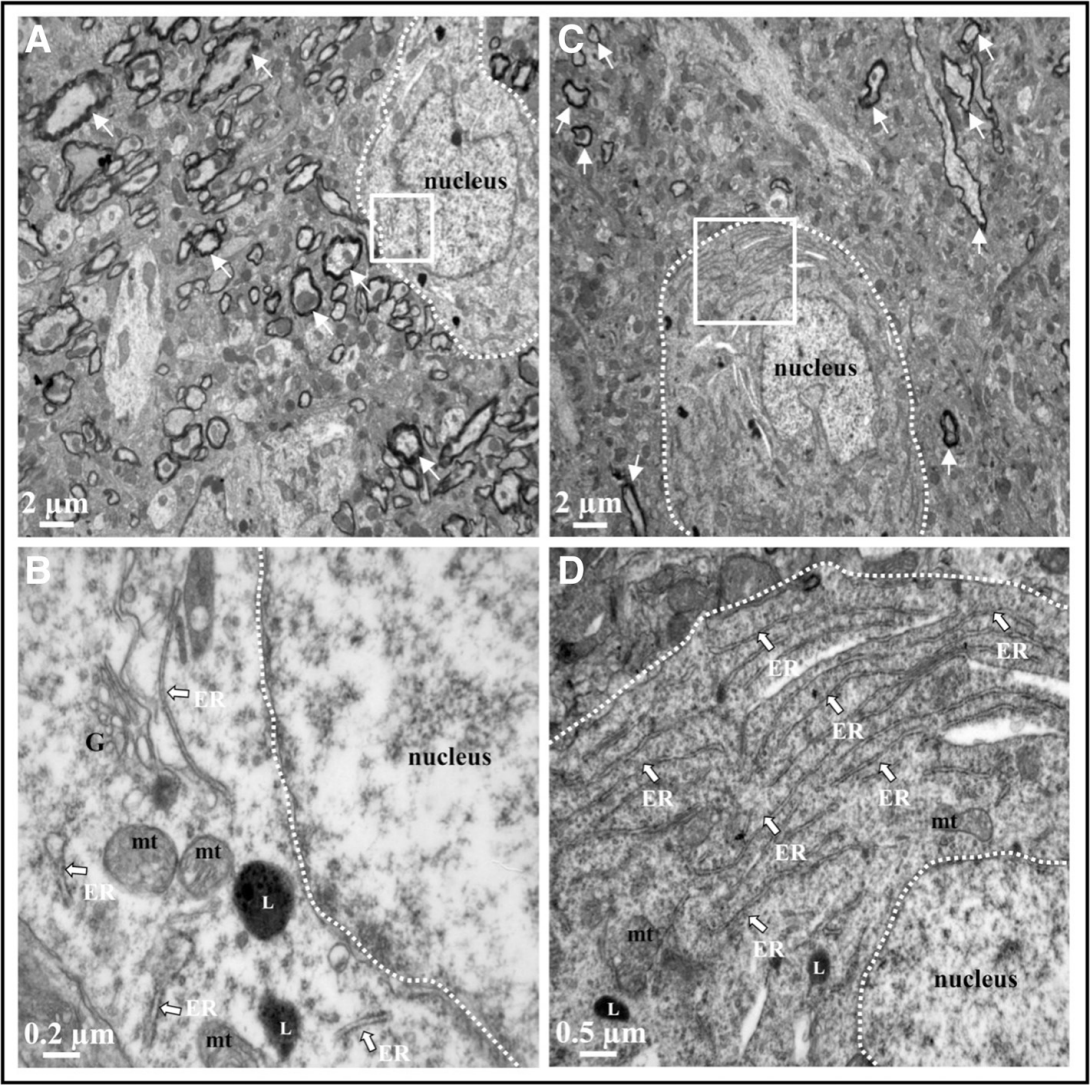
**Ultrastructure of cortex after acute LPS-induced inflammation.** TEM cortical images of 0.9% NaCl-injected mice **(A and B)** and 10 mg/kg LPS-treated mice (2 injections per 24 h, images **C** and **D**). No alteration of tissue integrity was observed in low magnification images **(A and C)**, myelin rounded axons marked with white arrow. Images **B** and **D** represent magnified region of interest of the white square in **A** and **C**, respectively. In these magnified images, mitochondria appeared with intact cristae (mt), endoplasmic reticulum (ER) was present with ribosomes and lysosomes (L) were observed. Five sections in each area (cortex and hippocampus) were observed for each mouse brain.

**Figure 4:**
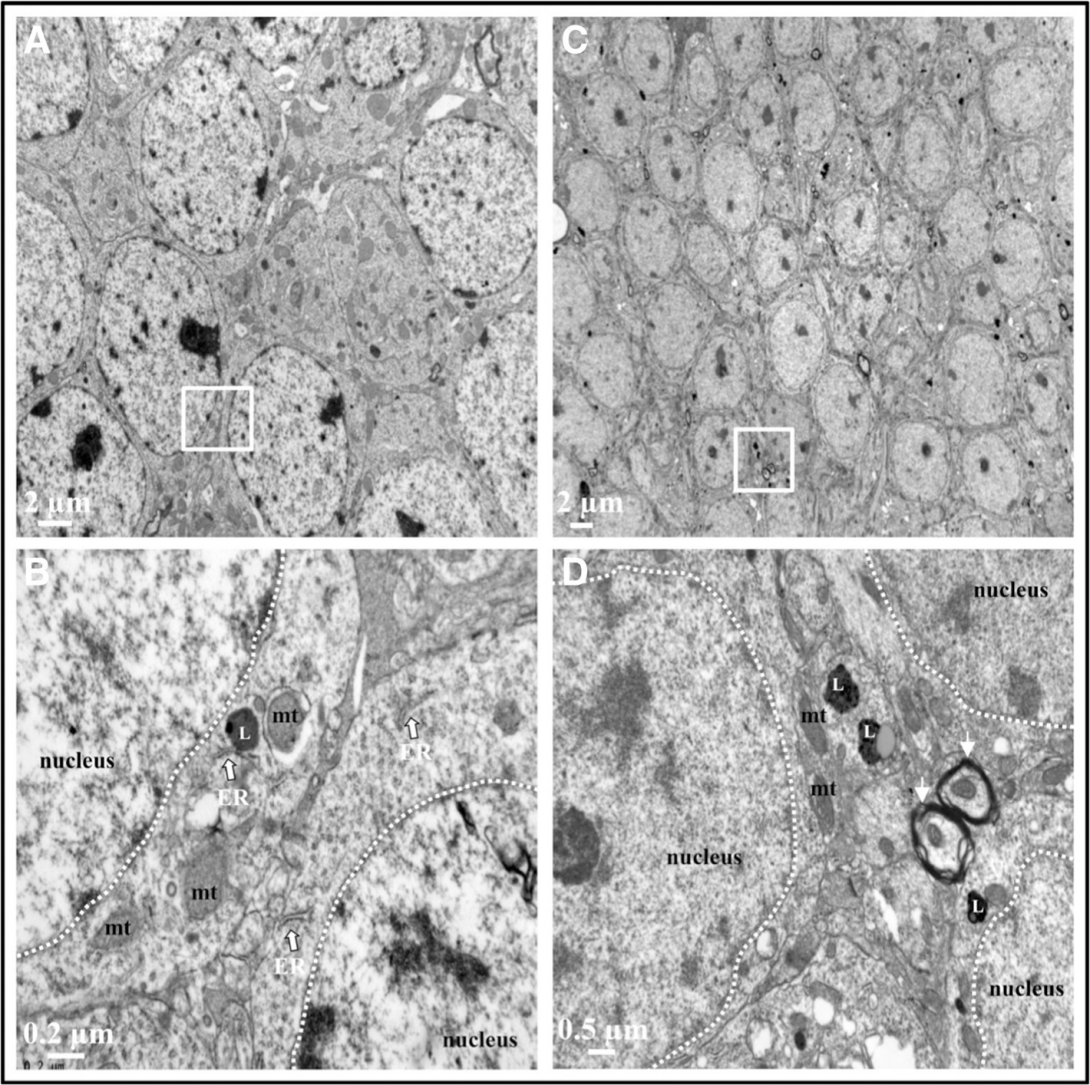
**Ultrastructure of hippocampus after acute LPS-induced inflammation.** TEM hippocampal images of 0.9% NaCl-injected mice **(A andB)** and 10 mg/kg LPS-treated mice (2 injections per 24 h, images **C** and **D**). No alteration of tissue integrity was observed in low magnification images **(A andC)**. Images b and d represent magnified region of interest of white square in a and c, respectively. In these magnified images, mitochondria appeared with intact cristae (mt), endoplasmic reticulum (ER) was present with ribosomes, myelin rounded axons marked with white arrow and lysosomes (L) were observed. Five sections in each area (cortex and hippocampus) were observed for each mouse brain.

Ultrastructure of cells in cortex and hippocampus after systemic LPS administration (two injections per 24 h) was similar to control mice with normal morphology of the mitochondria and the nucleus with evenly distributed chromatin is visible. Cytoplasm is rich in ribosomes and polyribosomes. No accumulation of vesicles was observed in cortex and in hippocampus (Figures 3 and 4).

### Activation of mTOR signalling pathway after acute LPS-induced inflammatory stress

mTOR activation leads to phosphorylation of various substrates, in particular p70S6K at T389, a ribosomal S6 kinase involved in ribogenesis [48],[49]. Furthermore, mTOR negatively regulates autophagy in several experimental models.

**Figure 5:**
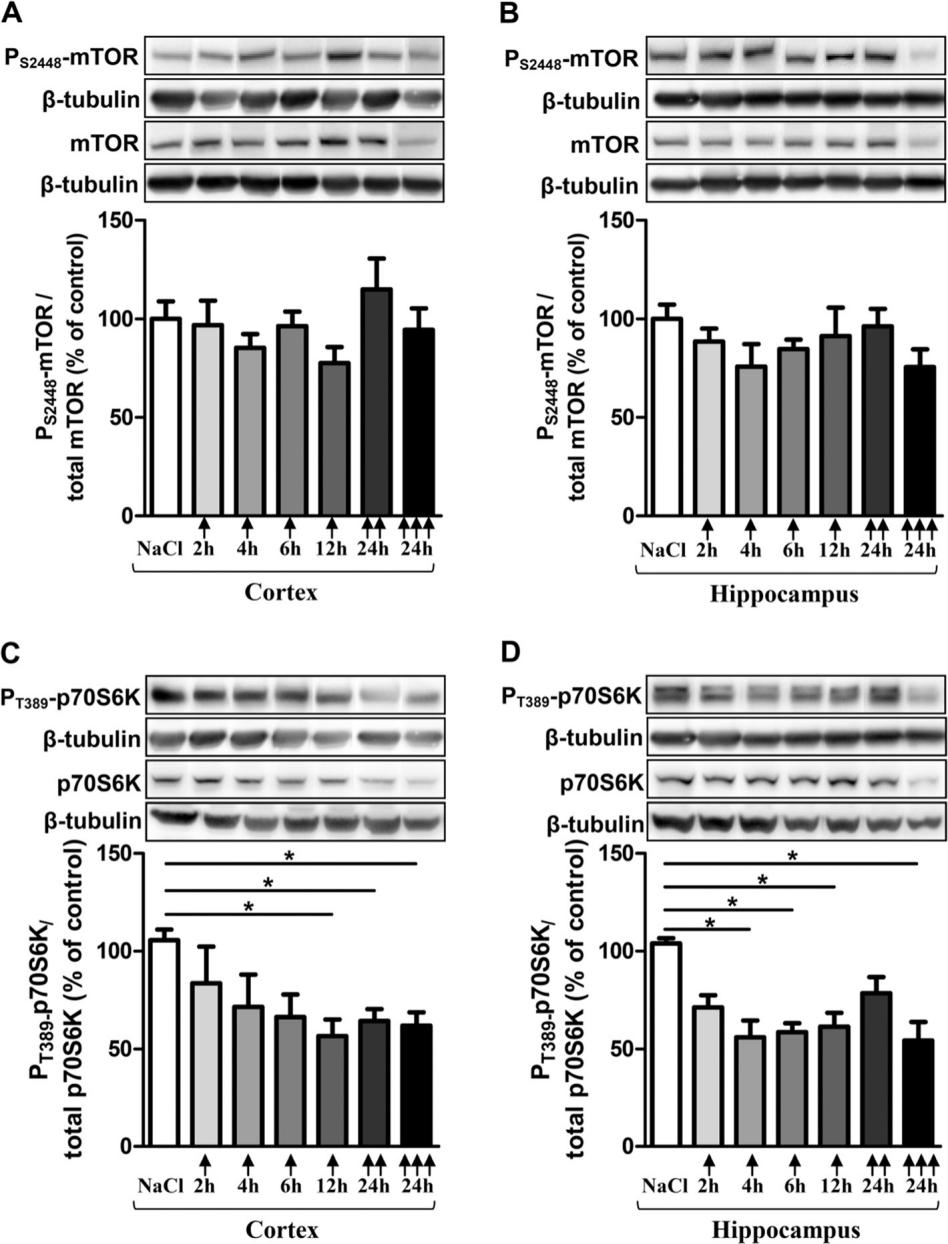
**Activation of the mTOR signalling pathway after acute LPS treatment.** Representative immunoblots showed the immunoreactivity of mTOR, P_S2448_-mTOR, p70S6K and P_T389_-p70S6K in cortex **(Aand C)** and in hippocampus **(Band D)** from treated-mice with a single () or two () or three () injections in i.p of LPS at 10 mg/kg or control (0.9% NaCl). Mice were sacrificed 2, 4, 6 or 12 h after a single injection and 24 h after two and three injections. Semi-quantitative analysis of immunoblot was performed using Gene Tools software (Syngene, Ozyme France). The immunoreactivity of protein was normalized to β-tubulin immunoreactivity. The results are expressed as arbitrary units (% of 0.9% NaCl-injected mice group set at 100%). Results are mean ± SEM for 6 mice in each group. *p < 0.01 compared to 0.9% NaCl-injected mouse group by Kruskal-Wallis test with a Dunns multiple comparison test.

No modification of the mTOR activation was observed after an acute LPS stress (Figure 5A and B). However, the p70S6K activation decreased in time-dependent manner and significantly from 12 h in the cortex (46%) and from 4 h in the hippocampus (46%) after one 10 mg/kg LPS injection (Figure 5C and D). With two and three injections of LPS per 24 h, the p70S6K activation decreased in both brain areas (24-48%). No statistically significant variations of the mTOR and p70S6K activation were observed in cortex and in hippocampus whatever the design of treatment in saline-injected group mice (see Additional file 1: Table S3 and S4). Therefore, results for NaCl correspond to the mean in each panel of Figure 5.

### Correlations between cytokine levels and expression of autophagic markers after acute LPS-induced inflammatory stress

In our experimental conditions, acute LPS treatment stimulated cytokine production (IL-1β, TNF-α and IL-6), decreased autophagic marker expressions and p70S6K activation without immediate cortical or hippocampal tissue damage as shown by TEM. Spearman correlations were performed between inflammatory and autophagic parameters. In cortex, a positive correlation between IL-6 levels and p70S6K expression was found (rho = 0.88; p = 0.03; n = 6 mice) at 12 h after a single LPS injection. In hippocampus and after two LPS injections per 24 h, two correlations were noted one between IL-1β levels and LC3 II expression (rho = 0.94; p = 0.01; n = 6 mice) and a second between TNF-α levels and p70S6K expression (rho = −0.88; p = 0.03; n = 6 mice).

In this first part, an acute peripheral inflammatory stress affected the cerebral autophagy with a positive correlation between LC3 II and IL-1β levels after two 10 mg/kg LPS injections per 24 h. Moreover, p70S6K expression significantly decreased and the levels of p70S6K were positively correlated to IL-6 levels in the cortex at 12 h and negatively to TNF-α levels in the hippocampus at 24 h with two i.p. LPS injections. In parallel, we also wanted to study the impact of a chronic inflammatory stress in autophagy. Three months old mice received an i.p. dose of 0.5 mg/kg of LPS every 3 days for 3 months. This treatment did not affect the life expectancy of mice compared to control mice (0.9% NaCl as vehicle). The average weight was 31.44 ± 5.63 and 34.53 ± 4.75 mg for control and LPS-treated mice, respectively.

### Pro-inflammatory cytokine levels after chronic LPS-induced inflammatory stress

**Figure 6:**
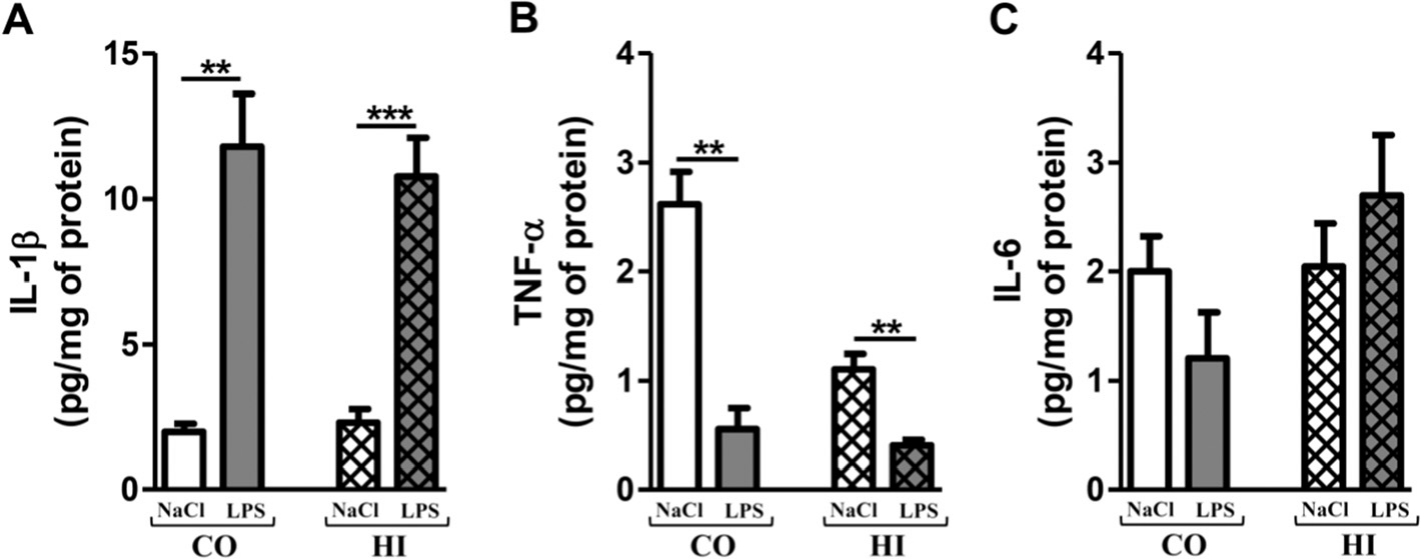
**Pro-inflammatory cytokine levels after chronic LPS-induced inflammatory stress.** IL1-β **(A)**, TNF-α **(B)** and IL-6 **(C)** levels in cortex (CO) and hippocampus (HI) from LPS-treated mice (0.5 mg/kg of LPS every 3 days for 3 months) or control (0.9% NaCl every 3 days for 3 months) by using ELISA assay. Treatment started at 3 months and mice were sacrificed at 6 months of age. Cytokine levels were expressed in pg/mg protein. Results are mean ± SEM for 6 mice in each group. ^**^p < 0.01, ^***^p < 0.001 compared to control mice by a Mann–Whitney test.

After 3 months of treatment, IL-1β levels significantly increased in cortex (495%) and in hippocampus (367%) compared to control mice (Figure 6A). Surprisingly, TNF-α levels decreased in both brain areas: 79% in cortex and 63% in hippocampus (Figure 6B). For IL-6, no difference was observed in LPS-treated mice versus control mice (Figure 6C).

### Changes in autophagy markers after chronic LPS-induced inflammatory stress

**Figure 7:**
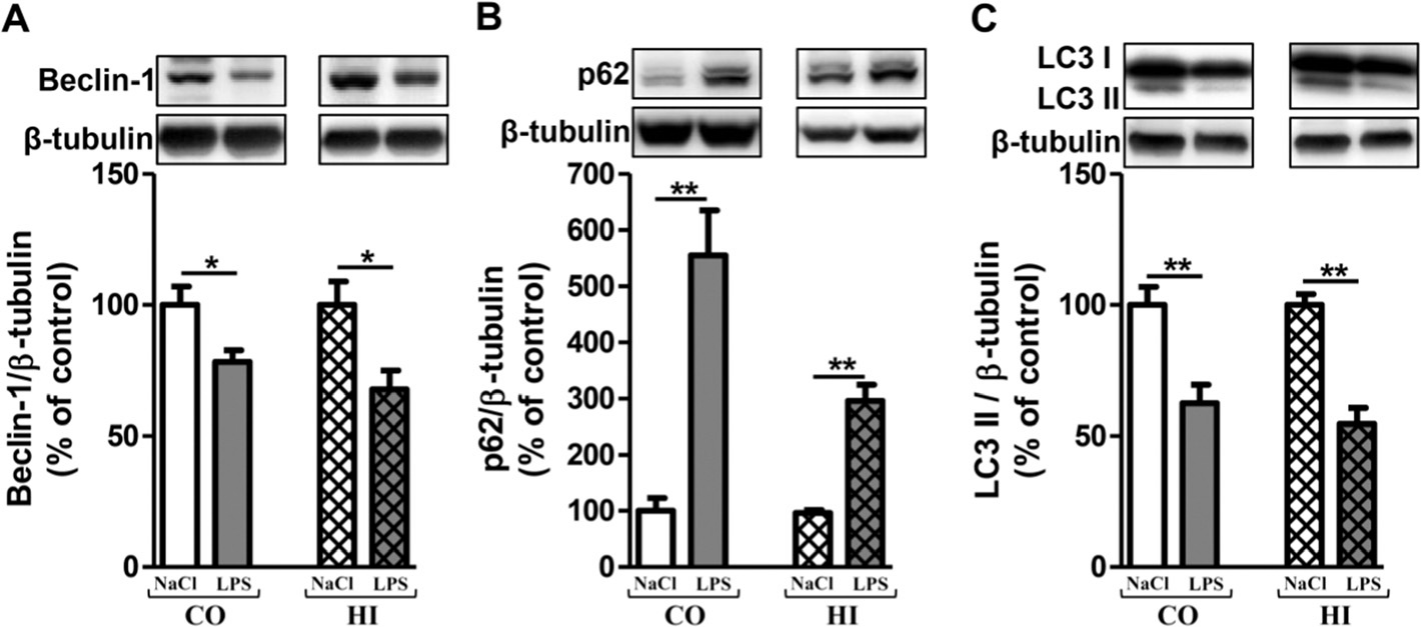
**Changes in autophagy markers after chronic LPS-induced inflammatory stress.** Representative immunoblots showed the immunoreactivity of Beclin-1 **(A)**, p62 **(B)** and LC3 II **(C)** in cortex and hippocampus from LPS-treated mice (0.5 mg/kg of LPS every 3 days for 3 months) or control (0.9% NaCl every 3 days for 3 months). Treatment started at 3 months and mice were sacrificed at 6 months of age. Densities were quantified by using Gene Tools software (Syngene, Ozyme France). Data of each protein were reported to data of the corresponding β-tubulin. The results are expressed as arbitrary units (% expression of 0.9% NaCl-treated mice set at 100%). Results are mean ± SEM for 6 mice in each group. ^*^p < 0.05, ^**^p < 0.01 compared to control mice by Mann–Whitney test.

Chronic LPS-induced inflammatory stress decreased Beclin-1 by 24% and 32% in cortex and hippocampus, respectively (Figure 7A). On the contrary, a robust increase of p62 expression was observed in both areas: 455% in cortex and 208% in hippocampus (Figure 7B). A significant decrease of LC3 II expression was observed in both areas (37.5% in cortex and 45% in hippocampus) without changes in LC3 I levels (Figure 7C).

**Figure 8:**
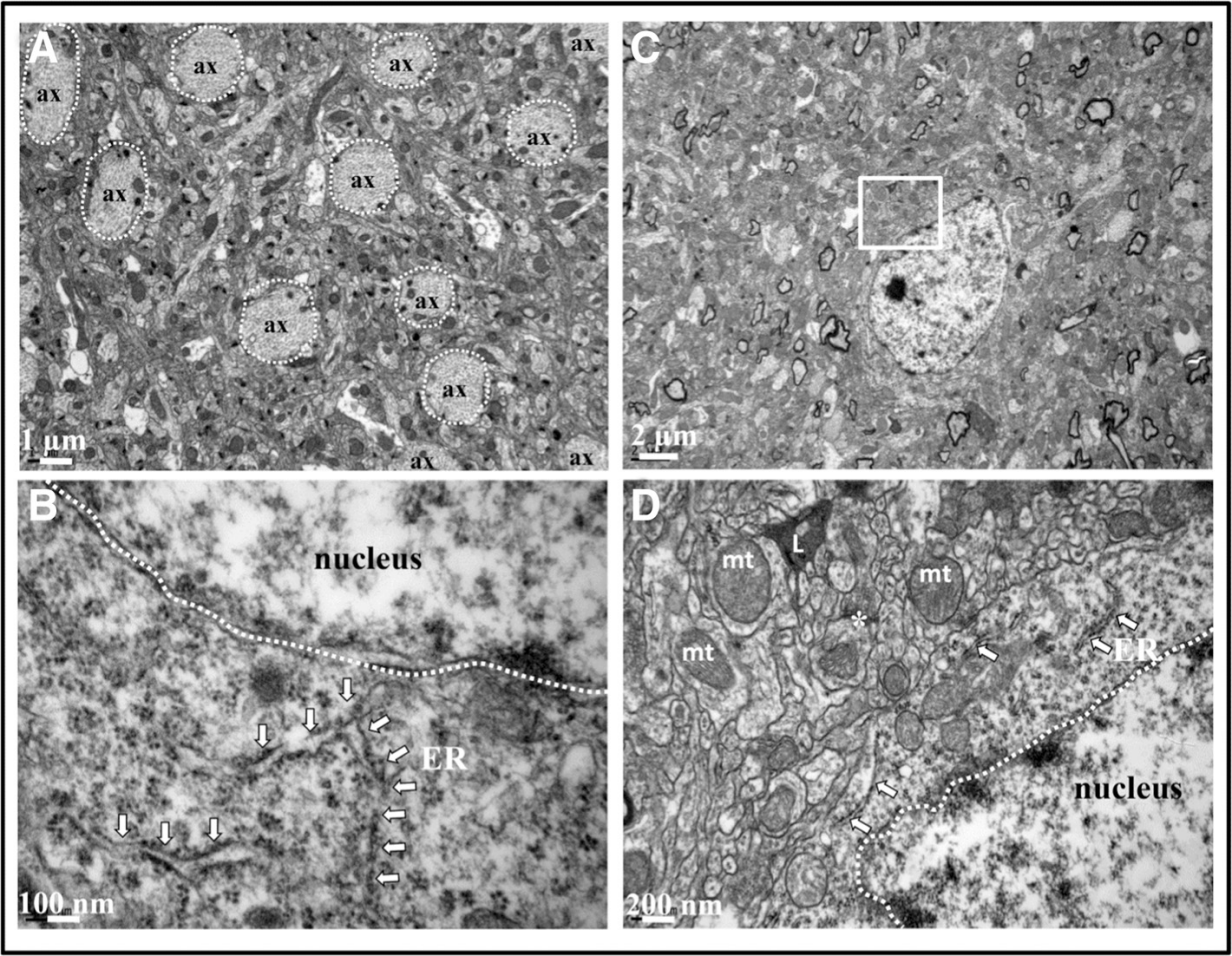
**Ultrastructure of cortex and hippocampus after chronic LPS-induced inflammatory stress.** TEM was performed in LPS-treated and control mice (n = 3 in each group) in cortex **(Aand B)** and in hippocampus **(Cand D)**. Low magnification image of cortex **(A)** showed a regular distribution of axons (ax) and microtubule filament inside. Image d represent magnified region of interest showed in the white square in **C**. The endoplasmic reticulum (ER) marked with white arrow **(B and D)** carried ribosomes without abnormalities. Mitochondria (mt) have intact cristae, lysosomes (L) were observed and synapses with pre-synaptic vesicles are marked by (*). Five sections in each area (cortex and hippocampus) were observed for each mouse brain.

Similarly to what was observed after acute LPS-induced inflammatory stress, TEM showed that chronic LPS-induced inflammatory stress did not cause major morphological tissue alterations. At the cellular level, mitochondria appeared with a normal shape without alteration and no vacuole accumulations were observed in cortex and in hippocampus (Figure 8).

### Activation of mTOR signaling pathway after chronic LPS-induced inflammatory stress

**Figure 9:**
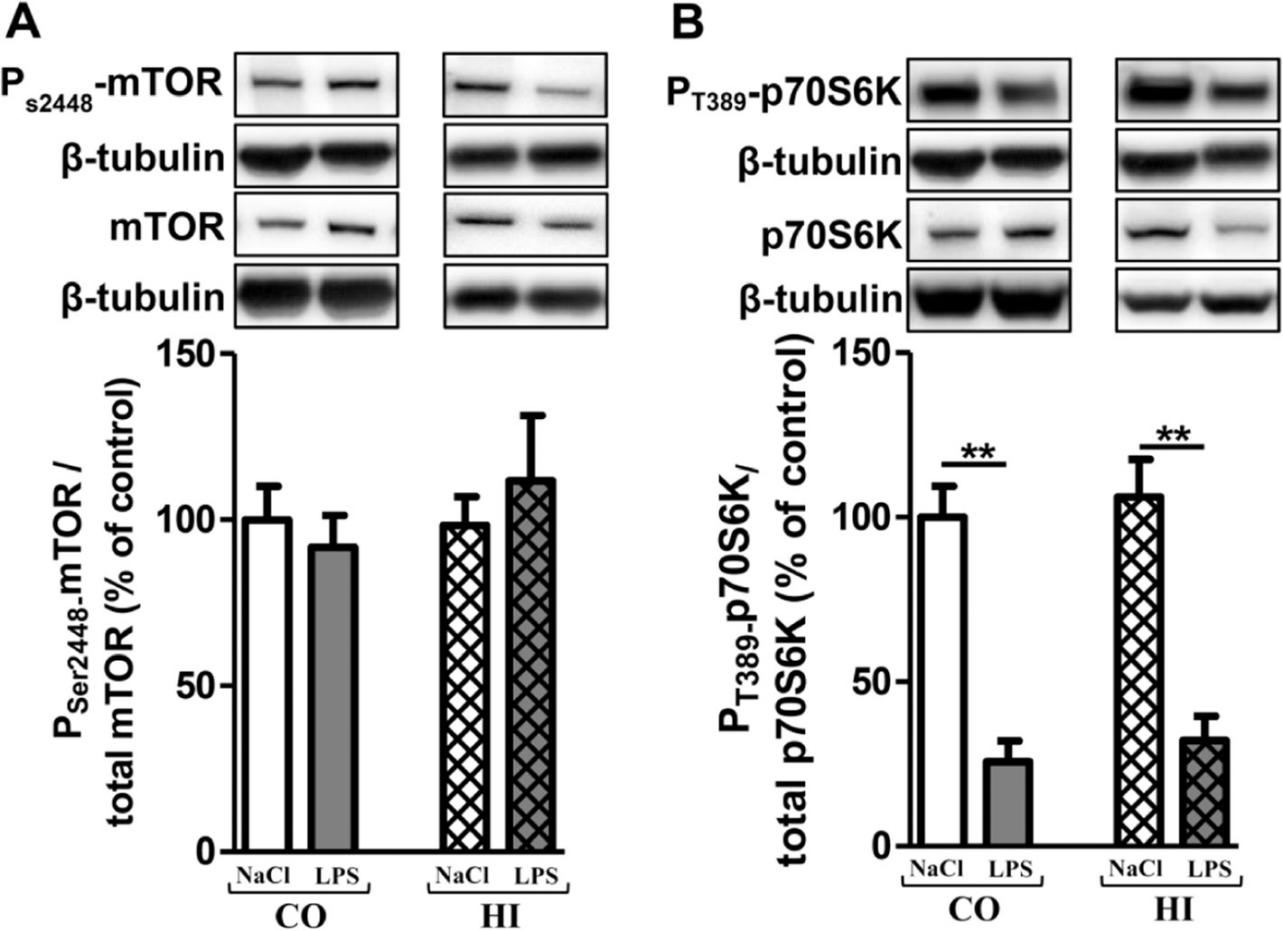
**Changes in mTOR and p70S6K activation after chronic LPS-induced inflammatory stress.** Representative immunoblots showed the immunoreactivity of mTOR, P_S2448_-mTOR, p70S6K, P_T389_-p70S6K in cortex **(A)** and in hippocampus **(B)** from LPS-treated mice (0.5 mg/kg of LPS every 3 days for 3 months) or control (0.9% NaCl every 3 days for 3 months). Treatment started at 3 months and mice were sacrificed at 6 months of age. Semi-quantitative analysis of immunoblot was performed using Gene Tools software (Syngene, Ozyme France). The immunoreactivity of protein was normalized to β-tubulin immunoreactivity. The results are expressed as arbitrary units ((% of 0.9% NaCl-injected mice group set at 100%). Results are mean ± SEM for 6 mice in each group. ^**^p < 0.01 compared to control mice by a Mann–Whitney test.

Similarly to acute LPS-induced inflammatory stress, the mTOR activation was not modified (Figure 9A). However, the p70S6K activation was dramatically reduced in cortex (75%) and hippocampus (74%) (Figure 9B).

### Correlations between levels of cytokine and autophagic markers after chronic LPS treatment

Chronic LPS treatment induced IL-1β production associated with changes of autophagic marker expressions and a great decrease of p70S6K activation without mTOR activation and without tissue morphological alterations. Interestingly, Spearman analysis of data from LPS-treated mice revealed two positive correlations between cortical IL-1β and Beclin-1 and between IL-1β and LC3 II levels. Furthermore, a negative correlation between IL-1β and p62 levels was observed (Table 3). In this LPS mouse group, only IL-1β would control cortical autophagy. Another interesting correlation revealed that levels of beclin-1 expression were negatively correlated with those of p62 (rho = −0.88, p = 0.03). This last correlation would reinforce that the cortical autophagic flux would be induced after a chronic and peripheral LPS treatment. However, any correlation was observed in hippocampus.

**Table 3:** Correlation between autophagic factors and IL-1β levels during chronic LPS –induced inflammatory stress

	**Beclin-1**		**p62**		**LC3 II**	
	**rho**	**p**	**rho**	**p**	**rho**	**p**
**IL-1β**	0.88	0.03	- 0.88	0.03	0.88	0.03

Spearman correlations were performed between IL-1β and autophagic parameters measured in the cortex of LPS-treated mice (0.5 mg/kg of LPS every 3 days for 3 months). Treatment started at 3 months and mice were sacrificed at 6 months of age (n = 6). In table, rho and p values were indicated. The level of significance was p < 0.05.

## Discussion

Alteration of autophagy and excessive inflammatory response are two hallmarks common to various brain diseases such as AD, PD, HD, ALS [13],[39]. However, the relationships between these two defense mechanisms of the body remain unknown in the CNS. Recently, we showed in vitro that IL-1β was involved in the acidic vesicle accumulation in microglia contrary to amyloid peptide [50]. The current study therefore aimed at determining whether an inflammatory reaction could modulate the autophagic process in the CNS.

The i.p. injection of LPS is extensively used to induce brain inflammation [40],[41],[51]. Several studies showed that treatment of mice with LPS induced a central inflammatory response associated with microglial activation, immunomodulatory effects of astrocytes, cyclo-oxygenase-2 and iNOS immunoreactivities and increases in cytokine productions [41],[42],[52]–[55].

However there are various regimens followed in mice. Among the acute treatments, the most frequently encountered are i.p. LPS injection of 0.5, 1 or 5 mg/kg and sacrifice of animals after one hour to ten days. In that issue, several studies only reported the transcriptional analysis of cytokines, in particular IL-1β, IL-6 and TNF-α in brain regions, showing an increase in mRNA expression of these cytokines in hippocampus, choroid plexus [51],[56]–[58]. Here, we used a higher dose (10 mg/kg) because lower LPS doses (i.e. single dose of 1 mg/kg or three doses of 3 mg/kg per 24 h) failed to detect significant increases in cytokine production in the mouse brains by using ELISA as previously reported [59]. In the present study, brain inflammatory response was reflected by a great increase in IL-6 levels started at 4 h in hippocampus and 6 h in cortex after a single injection while levels of IL-1β and TNF-α increased after two or three LPS injections per 24 h.

For the chronic LPS treatment, some authors included in their experimental design a group of mice received a single dose of LPS at 5 mg/kg or 10 mg/kg and sacrificed after 1, 3 or 10 months [41],[54]. Here, 0.5 mg/kg LPS was i.p. injected every three days during three months. Other authors chose twice injections per week for 6 weeks at a dose of 0.5 mg/kg in 4-month-old 3xTg-AD transgenic Alzheimer mouse model [60]. In last study, the monitoring of mRNA expression of IL-1β, IL-6 and TNF-α showed that IL-1β levels were markedly increased in the brains of LPS-treated mice. However, IL-6 and TNF-α expression levels were not significantly altered by LPS treatment. In our experimental conditions, chronically LPS-treated wild-type mice displayed a significant increase in IL-1β whereas TNF-α levels significantly decreased. Therefore, IL-1β could be considered as the critical cytokine in the central inflammatory response induced by peripheral LPS during 3 months. The decrease of TNF-α levels was also observed in brain, liver and serum after a long term time course of single 5 mg/kg LPS injection [54]. For this higher LPS dose, authors demonstrated a role of TNF receptors (TNFR) since in TNFR KO mouse models, LPS-induced TNF-α production was totally inhibited, suggesting a potential downregultion of these receptors in our experimental design. One may also propose that this decrease in TNF-α levels could be due to a modification of TNF-α converting enzyme (TACE) activity, also known as ADAM17 (a disintegrin and metalloprotease-family) and involved in the cleavage of TNF-α precursor to produce mature TNF-α [61]. In accordance with these data, we previously demonstrated that at 18 months of age, APPswePS1dE9 mice displayed a great decrease in TNF-α production [62].

Furthermore, the immuno-tolerance induced by chronic LPS injections could explain the decrease of TNF-α rate in cortex and in hippocampus and maintain a significant higher level of IL-1β. Indeed, it is well established that a first LPS exposure induced an overproduction of cytokines following a modification of gene expression in monocyte/macrophage becoming refractory to secrete some cytokines like TNF-α [48],[63],[64].

Despite this inflammatory response, normal tissue morphology and cell integrity were preserved in both acute and chronic LPS treatment. No signs of cellular damage were visible by TEM after both acute and chronic treatments. Authors showed that acute 1 mg/kg of LPS injection induced no neuronal death (negative Fluorojade B neurons and negative TUNEL neurons) and no rupture of blood brain barrier (BBB) [52],[65]. In addition, maintenance of the mitochondrial architecture critically depends on the induction of autophagy which is essential for regenerating astrocyte mitochondrial networks during inflammation [66]. But ultrastructure of a neuronal cell in the hippocampus 48 h after 1 mg/kg LPS administration showed shrunken and dark cytoplasm, deep invagination of the nuclear envelope into the nucleoplasm and swelling of some mitochondria [51].

The monitoring of autophagy markers, including Beclin-1, p62 and LC3, in mouse brains after systemic LPS-induced inflammatory stress, has never been conducted before. Only two papers described an increased expression of lysosomal cysteine proteases, cathepsins (Cat) C and B [51],[56]. Cat C expression was detected in neurons of cerebral cortex 6 h after 5 mg/kg LPS i.p. injection and 24 h later, Cat C expression was also detected in activated microglial cells throughout the entire brain. The duration of induced Cat C expression in neurons and in microglial cells was ten days and three days, respectively by using in situ hybridation (ISH) and immunohistochemical staining (IHC) [56]. An immunocytochemical analysis of the subcellular localisation of Cat B using post-embedding immunogold methods showed that, 48 h after systemic 1 mg/kg LPS administration, Cat B was translocated from lysosomes to the cytosol and autophagic vacuoles and was also found in the membrane of mitochondria in the hippocampal area.

Our study showed that acute 10 mg/kg LPS treatment induced autophagy early changes in the hippocampus with increased p62 and decreased LC3 II 4 h after injection evolving towards a decrease in the three parameters (Beclin-1, p62 and LC3 II) 12 h after injection. In the cortex, the LC3 II decrease was observed from 12 h. After 24 hr, all markers of autophagy significantly decreased in the cortex and hippocampus. Interestingly, in hippocampus IL-1β levels were positively and strongly (rho = 0.94) correlated to LC3 II expression, indicating a role of this cytokine in LC3 II induction. Several cytokines, including IL-1β are well known as autophagy stimulatory molecules [67]–[69]. Furthermore, the induction of autophagy was associated with a great inactivation of p70S6K (or Ribosomal S6 Kinase 1) without modification of mTOR activation. The T389 site of p70S6K is known to be phosphorylated by the kinase mTOR. However, extensive research on the regulation of the activity of p70S6K studies show direct control by the active Receptor Tyrosine Kinase (RTK)/Phosphoinositide 3-kinase/Phosphoinositide-dependent kinase-1 (PDK1) signaling pathway that the phosphorylation site is not defined yet [48]. In addition, RS6K1 dephosphorylation was an active process of its regulation by protein phosphatase 2 (PP2A) [70],[71]. Here, a negative correlation was observed between p70S6K activation and TNF-α levels in hippocampus, indicating the negative impact of LPS-induced sepsis in this kinase linked to diverse cellular processes, including protein synthesis, mRNA processing, glucose homeostasis, cell growth and survival. Other authors showed that an in vivo sepsis not induced by LPS inhibits mTOR signaling pathways in rat cardiac muscle and that this defect appeared mediated, either directly or indirectly, by the endogenous over production of TNF-α [72].

Based on the results obtained, a differential expression level of autophagy markers between in cortex and in hippocampus was mainly observed after one injection of LPS. Hippocampus responded faster than cortex in particular for Beclin-1, LC3 II and p70S6K. These differences of autophagy levels between these two brain structures were also observed in rats after various acute stresses such as hypoxia-ischemia, oxygen and glucose deprivation or 6-OHDA injection [73]–[75]. Some authors showed a differential brain activity after a peripheral inflammation. It was demonstrated that during sepsis, the BBB lose its structural integrity allowing the cross of peripheral cytokines and macrophages in the brain [76],[77]. The brain can be sense peripheral inflammation through the vagus nerve and the hypothalamic–pituitary–adrenal axis and influence the brain activity, memory, plasticity, neurogenesis [78]. These brain-peripheral immune interactions could also explain the differential expression level of autophagy markers between in cortex and in hippocampus in our study.

Although some authors have examined the level of expression of cathepsins after acute treatment with LPS in mice, no study has yet been published on the autophagic changes in long-term chronic treatment with LPS. This work, however, is very important when taken into consideration the fact that many neurodegenerative diseases are characterized by disturbances both in the regulation of inflammation and autophagy. We observed after 3 months of neuroinflammation induction, an upregulation of p62 coupled to a net decrease in expression of Beclin-1 and LC3 II. Very interestingly, correlation analysis revealed that the IL-1β production after chronic LPS treatment induced autophagic flux. In accordance with our results, it is known that inflammasome, in particular caspase 1 also increases autophagic flux [79]. However, a recent study revealed that inflammatory stimulus in macrophage cell line and in human macrophages activated autophagy and decreased production of IL-1β production [26] due to the degradation of inflammasome complex by autophagy. This feedback would be necessary to counteract and to limit the inflammation reaction due to minor insult. In our in vivo study, this beneficial autophagy feedback could explain that one LPS injection was not sufficient to measured significant IL-1β levels in cortex and hippocampus and required more injections (2 or 3 i.p injections). Interestingly, a chronically administration but not a lower LPS dose could impair the beneficial autophagy feedback against IL-1β production and induce an increase of IL-1β levels in cortex and in hippocampus.

Regarding p62, results showed negative correlation between its levels of expression and those of IL-1β. In other experimental conditions, recent findings showed that the production and secretion of the proinflammatory cytokine IL-1β was significantly enhanced in p62−/− macrophages after infection with *Legionella pneumophila*. Furthermore, these authors showed that p62 may interact with nucleotide-binding oligomerization domain-like receptor (NLR) family, CARD domain-containing 4 and NLR family, pyrin domain-containing 3 proteins to inhibit their self-dimerization [80]. However, its self-dimerization is a necessary step for its degradation during autophagy [81]. Based on these physical interactions, p62 could accumulate as we observed after a chronic LPS treatment.

## Conclusion

An acute and a chronic peripheral inflammatory stress induced by LPS, in particular, with a persistent IL-1β production modified cortical and hippocampus autophagic marker expressions. Chronic inflammatory stress increased p62 and decreased Beclin-1, LC3 II and p70S6K activation without changes of the mTOR activation and any morphological tissue alteration. Moreover, IL-1β levels were positively correlated to Beclin-1 and LC3 II while p62 expression was inversely correlated to IL-1β levels after chronic neuroinflammation. These findings highlighted the induction of central autophagy by IL-1β-mediated inflammation. It is important to note that the rate of this inflammatory factor remains very moderate, less than 15 pg/mg protein in both brain regions studied and therefore this level would be interesting to activate autophagy in neuroinflammatory diseases including neurodegenerative diseases characterized by a great inflammation and accumulation of autophagosomes in advanced stages.

## Methods

### Chemical products

Sodium fluoride (NaF), phenylmethylsulfonyl fluoride (PMSF), protease and phosphatase inhibitor cocktails, dithiothreitol (DTT), Lipopolysaccharide (LPS), Paraformaldehyde (PFA) and all reagent-grade chemicals for buffers were purchased from Sigma (St Quentin Fallavier, France); Sodium pentobarbital from CEVA, Animal Health (Libourne, France); NuPAGE® LDS 4X LDS Sample Buffer, NuPAGE® Sample Reducing Agent (10X), Novex® 4-20% Tris-glycine Mini gels, NuPAGE® 3-8% Tris-Acetate gels, Novex® Tris-Glycine SDS Running and NuPAGE® Tris-Acetate SDS running buffers, NuPAGE® Antioxidant, Seeblue® Plus2 pre-stained standard, iBlot® Gel Transfer Device (EU), Quant-it® protein assay from Gibco-Invitrogen (Fisher Bioblock Scientific distributor, Illkirch, France); 4X Laemmli sample buffer, 4-15% mini-PROTEAN® TGX™ gels, Tris-glycine running buffer and Trans-Blot® Turbo™ Transfer System from Biorad (Marnes-la-Coquette, France).

For western blot, primary antibodies and secondary anti-rabbit IgG antibody conjugated with Horseradish Peroxydase (HRP) were purchased from Cell Signalling (Ozyme, St Quentin Yvelines, France) excepted p62/SQMT1 from MBL (CliniSciences distributor, Nanterre, France), anti-β tubulin from Sigma (St Quentin Fallavier, France), peroxidase-conjugated anti-mouse IgG from Amersham Biosciences (Orsay, France), IgG- and protease-Free Bovine Serum Albumin (BSA) from Jackson ImmunoResearch Europe Ltd (Interchim distributor, Montluçon, France).

### Animals

Adult male and female B6C3F1 mice (3 months, 30.50 ± 0.82 mg in weight) were purchased from Charles River Laboratories (L’Arbresle, France). The use of animals for this study has received the approval of the Ethical and Animal Care Committee at “La direction départementale de la protection de la population (DDPP)” (registration number: 06.12). All animal cares and experimental procedures were conducted in conformity with the French Décret n° 2013–118 1st February 2013 NOR: AGRG1231951D in accordance with European Community guidelines (directive 2010/63/UE for the Care and Use of Laboratory Animals). All efforts were made to minimize animal suffering, as well as, the number of animals used. The animals were housed in a conventional state under adequate temperature (23 ± 3°C) and relative humidity (55 ± 5%) control with a 12/12 h reversed light/dark cycle, and provided with free access to food and water.

### Lipopolysaccharide-induced inflammatory stress

LPS (*Escherichia coli*, serotype 0111:B4) was used to induce an inflammatory response. Two experimental designs were performed. First, an acute treatment with LPS intraperitoneally (i.p.) injected at a dose of 10 mg/kg dissolved in sterile-endotoxin-free 0.9% saline vehicle. Control injections were equivolume vehicle. Mice (6 per group) were sacrificed after 2, 4, 6, 12 h post-injection. Two other groups received either two or three i.p. LPS injections per 24 h before sacrifice (n = 6 in each group). Second a chronic treatment with LPS consisted of an i.p. injection at a dose of 0.5 mg/kg every three days during three months (6 controls versus 6 LPS mice). For this chronic treatment, mice were weighted once a week. The dosage of LPS in both acute and chronic treatments was based on previous studies of LPS-neurotoxicity [52],[54],[82]. For scanning electron microscopy, three mice per group were also included in this study.

### Brain tissue preparation

LPS-treated and control (0.9% saline vehicle) mice were transcardially perfused with phosphate buffer saline (154 mM NaCl, 1.54 mM KH_2_PO_4_, 2.7 mM Na_2_HPO_4_.7H_2_O, pH 7.3) after deep anesthesia with pentobarbital (80 mg/kg, i.p.). Brains (6 per group for biochemical assays) were rapidly removed and dissected on ice. Cortex and hippocampus were homogenized using 10 up-and-down strokes of a prechilled Teflon-glass homogenizer in 20 volumes of lysis buffer (25 mM Tris–HCl, 150 mM NaCl, 1 mM EDTA, pH 7.4) and supplemented with 50 mM NaF, 1 mM PMSF, protease and phosphatase inhibitor cocktails (50 μL/gr of tissue and 10 μL/mL of lysis buffer, respectively). Lysates were sonicated and centrifuged at 15,000 *g* for 15 min at 4°C. The resulting supernatants were collected and protein concentrations were determined using Quant-it® protein assay according to the manufacturer’s protocol. Samples were stored at −80°C until ELISA and immunoblotting described below.

### Cytokine Enzyme-linked immunosorbent assay (ELISA)

Commercially available ELISA kits were used for measuring mature form of IL-1β (sensitivity: 16 pg/mL), TNF-α (sensitivity: 4 pg/mL) and IL-6 (sensitivity: 2 pg/mL) according to the manufacturers’ instructions (BioLegend, Ozyme distributor, St Quentin Yvelines, France). The range of analysis was between 31.3-2,000 pg/mL for IL-1β and 7.8-500 pg/mL for TNF-α and IL-6. Homogenates from brain tissue (50 mg of tissue/mL) were added in each well of pre-coated plates and all steps were performed at room temperature (RT). The enzymatic reaction was stopped after 15 min incubation with tetramethylbenzidine (TMB) substrate by adding 2 N H_2_SO_4_ and the optical density (OD) was read at 450 nm within 30 min, using Multiskan® spectrum spectrophotometer. The cytokine levels were then calculated by plotting the OD of each sample against the standard curve. The intra- and inter-assay reproducibility was >90%. OD values obtained for duplicates that differed from the mean by greater than 10% were not considered for further analysis. For convenience, all results are expressed in pg/mg protein.

### Immunoblotting

Samples (40 μg proteins) were prepared for electrophoresis by adding NuPAGE® 4X LDS sample buffer and NuPAGE® Sample Reducing Agent (10X). Samples were then heat-denatured at 100°C for 5 min, loaded into Novex® 4-20% Tris-Glycine mini Gels, run at 150 V for 60 minutes in Novex® Tris-Glycine SDS Running Buffer and in NuPAGE® 3-8% Tris-Acetate Gels, run at 125 V for 120 minutes in NuPAGE® Tris-Acetate SDS running buffer containing NuPAGE antioxidant. Gels were transferred to nitrocellulose membranes using the iBlot® Dry blotting system set at 20 V for 7 min. For LC3 immunoblot, we used Trans-Blot® Turbo™ Transfer System (25 V, 3 min for 0.2 μm nitrocellulose MISI format) after protein gel electrophoresis of samples prepared in 4X Laemmli sample buffer and loaded into 4-15% mini-PROTEAN® TGX™ gels with Tris-glycine SDS running buffer.

Membranes were washed for 10 min in Tris-buffered saline/Tween (TBST: 20 mM Tris–HCl, 150 mM NaCl, pH 7.5, 0.05% Tween 20) and aspecific antigenic sites were blocked by incubating the membranes in TBST containing 5% BSA for 2 h.

Blots were incubated with primary antibody in blocking buffer overnight at 4°C. Antibodies used were rabbit anti-P_S2448_-mTOR, anti-total mTOR, anti-P_T389_-p70S6K, anti-total p70S6K, anti-Beclin-1, anti-p62, anti-LC3, all at 1:500 dilution factor. Membranes were washed twice with TBST and then incubated with the HRP-conjugated secondary anti-rabbit IgG antibody (1:1000), during 1 hour at RT. Membranes were washed again and exposed to the chemiluminescence Luminata Forte Western HRP Substrate (Millipore, Saint-Quentin-en-Yvelines, France) followed by signal’s capture with the Gbox system (GeneSnap software, Syngene, Ozyme distributor). After 2 washes in TBST, membranes were probed with mouse antibody against tubulin (1:10000) overnight at 4°C. They were then washed with TBST, incubated with HRP-conjugated secondary antibody anti-mouse (1:1000) for 1 h, exposed to the chemiluminescence Luminata classico substrate (Millipore, Saint-Quentin-en-Yvelines, France) and signals were captured. Automatic image analysis software is supplied with Gene Tools (Syngene, Ozyme distributor). *Ratios* protein/tubulin were calculated and showed in the corresponding figures. *Ratios* Phospho-protein/total protein were calculated to evaluate rates of protein activation.

### Transmission electron microscopy (TEM)

Three mice in each group were were deeply anesthetized with pentobarbital (80 mg/kg, i.p.) and transcardially perfused with phosphate buffer saline (PBS: 154 mM NaCl, 1.54 mM KH_2_PO_4_, 2.7 mM Na_2_HPO_4_.7H_2_O, pH 7.2) and then with paraformaldehyde (PFA 4%). Brains were rapidly removed on ice and thin sagittal sections were isolated and fixed with 3% glutaraldehyde in phosphate buffer saline (0.1 M PBS; pH = 7.4) for 2 h at 4°C. Samples (2 mm^3^ of tissue in cortex and hippocampus) were then washed three times (3x10 min) in PBS and then post-fixed in 1% osmium tetroxyde in PBS for 1 h at 4°C, processed through a graded acetone series, embedded in Araldite (Fluka, Buchs, Switzerland) and polymerized overnight at 60°C. Thin sections (60 nm) were cut with a diamond knife on Reichert Ultracut S, recovered on Cu grids and contrasted with uranyl acetate (4%) and lead citrate and were observed under a JEOL 1010 transmission electron microscope (Jeol Ltd, Tokyo, Japan). 5 sections in each area (cortex and hippocampus) were observed for each mouse brain.

### Statistical analysis

For biochemical analysis, results were expressed as means ± SEM. To compare the two groups of mice in chronic treatment (control versus LPS mice) a mann-Withney’s test was used. Data for multiple variable comparisons were analyzed by a Kruskal-Wallis test with a Dunns multiple comparison test. For correlations between two parameters, we used a Spearman test (GraphPad Instat, GraphPad Software, San Diego, CA, USA). The level of significance was p < 0.05.

## Abbreviations

AD: Alzheimer’s disease

ADAM17: A disintegrin and metalloproteinase with a metallopeptidase domain 17

ALS: Amyotrophic lateral sclerosis

AMPK: Adenosine monophosphate kinase

Atg: Autophagy related genes

BBB: Blood brain barrier

BSA: Bovine Serum Albumin

CARD: Caspase recruitment domain

Cat: Cathepsin

CNS: Central nervous system

DTT: Dithiothreitol

ELISA: Enzyme-linked immunosorbent assay

ER: Endoplasmic reticulum

GWAS: Genome-wide association study

HD: Huntington’s disease

HRP: Horseradish peroxidase

i.p.: Intraperitoneal

IFN-γ: Interferon gamma

IHC: Immunohistochemical staining

iNOS: Inducible nitric oxide synthase

IL: Interleukin

ISH: In situ hybridation

JNK: Jun kinase

LC3: Microtubule-associated protein1 light chain 3

LDS: Lithium dodecyl-sulfate

LPS: Lipopolysaccharide

mt: Mitochondria

mTOR: Mammalian target of rapamycin

NaF: Sodium fluoride

NF-κB: Nuclear factor-kappa B

NLR: Nucleotide oligomerization domain receptors

OD: Optical density

PBS: Phosphate buffer saline

p62/SQMT1: Sequestosome 1

PD: Parkinson’s disease

PFA: Paraformaldehyde

PI3K: Phosphatidylinositide 3-kinase

PMSF: Phenylmethylsulfonyl fluoride

PP2A: Protein phosphatase 2A

RT: Room temperature

SDS: Sodium dodecyl-sulfate

TACE: TNFα converting enzyme

TBST: Tris-buffered saline tween

TEM: Transmission electron microscopy

TMB: Tetramethylbenzidine

TNF: Tumor necrosis factor

TNFR: Tumor necrosis factor receptor

## Competing interests

The authors declare that they have no competing interests.

## Authors’ contributions

AF performed the research, analyzed the data, their statistical significance and wrote the paper; FT participated in the design of the study and followed the work; NQ and BF carried out TEM; DC participated in brain tissue preparation for biochemical analysis; TJ, ARB and MP followed the research and provided relevant remarks throughout the work; GP conceived of the study, and organized its design and coordination and helped to draft the manuscript. All authors read and approved the final manuscript.

## Additional file

## Supplementary Material

Additional file 1: Table S1. Cortical cytokine levels in saline-treated mice. **Table S2.** Hippocampal cytokine levels in saline-treated mice. **Table S3.** Changes in cortical autophagic markers and mTOR signalling pathway in saline-treated mice. **Table S4.** Changes in hippocampal autophagic markers and mTOR signalling pathway in saline-treated mice. **Figure S1.** Accumulation of autophagic vesicles in murine primary mixed cell culture. Click here for file
