# Supplementary material for: Impairment of autophagy in the central nervous system during lipopolysaccharide-induced inflammatory stress in mice
Source: Mol Brain. 2014 Aug 27;7:56. doi: 10.1186/s13041-014-0056-z (PMC4237961; doi:10.1186/s13041-014-0056-z)
Supplement: Additional file 1: Table S1. — Cortical cytokine levels in saline-treated mice. Table S2. Hippocampal cytokine levels in saline-treated mice. Table S3. Changes in cortical autophagic markers and mTOR signalling pathway in saline-treated mice. Table S4. Changes in hippocampal autophagic markers and mTOR signalling pathway in saline-treated mice. Figure S1. Accumulation of autophagic vesicles in murine primary mixed cell culture. [file s13041-014-0056-z-S1.pdf]

Supporting Information

Table 1: Cortical cytokine levels in saline-treated mice

	Cortex					
	2h (↑)	4h (↑)	6h (↑)	12h (↑)	24h (↑↑)	24h (↑↑↑)
**IL-1β**	8.91 ± 3.26	7.54 ± 2.86	8.21 ± 2.43	9.33 ± 2.28	9.08 ± 2.90	8.19 ± 2.20
**TNF-α**	2.18 ± 0.28	3.06 ± 0.13	4.67 ±0.17	3.99 ± 0.61	2.17 ± 0.45	3.06 ± 0.13
**IL-6**	3.06 ± 0.65	1.88 ± 0.15	3.06 ±0.65	1.87 ± 0.15	2.21 ± 0.47	2.92 ± 0.51

Levels of IL-1β, TNF-α and IL-6 measured by ELISA in cortex of 3-months old mice treated either with one (↑) or two (↑↑) or three (↑↑↑) i.p. injections of 0.9% NaCl per 24h. Cytokine levels are expressed in pg/mg of protein. Results are ± SEM of 6 mice per group.

Table 2: Hippocampal cytokine levels in saline-treated mice

	Hippocampus					
	2h (↑)	4h (↑)	6h (↑)	12h (↑)	24h (↑↑)	24h (↑↑↑)
**IL-1β**	11.17 ± 1.60	10.00 ± 0.73	11.15 ± 1.61	10.02 ± 0.73	14.26 ± 0.22	11.67 ± 1.30
**TNF-α**	6.83 ± 0.28	5.38 ± 0.67	6.83 ± 0.27	5.37 ± 0.67	4.83 ± 0.53	5.09 ± 0.65
**IL-6**	4.95 ± 0.64	4.05 ± 0.92	3.50 ± 0.43	4.52 ± 0.31	2.79 ± 0.39	4.11 ± 1.55

Levels of IL-1β, TNF-α and IL-6 measured by ELISA in cortex of 3-months old mice treated either with one (↑) or two (↑↑) or three (↑↑↑) i.p. injections of 0.9% NaCl per 24h. Cytokine levels are expressed in pg/mg of protein. Results are ± SEM of 6 mice per group.

Table 3: Changes in cortical autophagic markers and mTOR signalling pathway in saline-treated mice

	Cortex					
	2h (↑)	4h (↑)	6h (↑)	12h (↑)	24h (↑↑)	24h (↑↑↑)
**P_{S2248}-mTOR/mTOR**	100.2 ± 8.8	95.7 ± 5.2	88.8 ± 7.7	105.0 ± 7.2	99.3 ± 6.8	92.5 ± 8.5
**P_{T389}-p70S6K/p70S6K**	105.4 ± 7.1	96.8 ± 7.7	113.0 ± 3.9	95.6 ± 7.6	95.2 ± 7.3	102.0 ± 9.3
**Beclin-1**	96.6 ± 5.3	100.5 ± 8.4	105.1 ± 4.8	95.2 ± 6.7	106.6 ± 6.5	97.0 ± 5.7
**p62**	107.5 ± 3.9	93.4 ± 3.6	105.1 ± 8.2	90.6 ± 3.2	101.4 ± 4.7	96.6 ± 5.4
**LC3 I**	100.0 ± 3.9	107.7 ± 3.5	98.0 ± 4.1	105.9 ± 4.7	108.0 ± 7.1	99.8 ± 4.2
**LC3 II**	106.2 ± 4.3	105.8 ± 4.5	102.5 ± 4.6	105.3 ± 1.3	115.8 ± 5.3	108.7 ± 3.7

Changes in autophagic markers Beclin-1, p62, LC3 I, LC3 II and in the activation of mTOR and p70S6K in cortex of 3-months old mice treated either with one (↑) or two (↑↑) or three (↑↑↑) i.p. injections of 0.9% NaCl per 24h. Semi-quantitative analysis of immunoblots was performed using Gene Tools software (Syngene, Ozyme France). The immunoreactivity of protein was normalized to β-tubulin immunoreactivity. The results are expressed as arbitrary units (%). Results are mean ± SEM for 6 mice in each group.

Table 4: Changes in hippocampal autophagic markers and mTOR signalling pathway in saline-treated mice

	Hippocampus					
	2h (↑)	4h (↑)	6h (↑)	12h (↑)	24h (↑↑)	24h (↑↑↑)
**P_{S2248}-mTOR/mTOR**	93.2 ± 9.3	98.4 ± 5.5	101.9 ± 4.8	96.4 ± 5.8	100.6 ± 6.4	104.1 ± 3.9
**P_{T389}-p70S6K/p70S6K**	94.2 ± 4.1	100.0 ± 6.2	96.7 ± 9.2	96.2 ± 8.4	102.4 ± 3.7	104.0 ± 8.8
**Beclin-1**	100.4 ± 3.8	99.7 ± 4.6	105.3 ± 7.1	98.3 ± 8.7	93.6 ± 4.7	100.7 ± 6.1
**p62**	99.5 ± 6.6	108.0 ± 4.9	102.0 ± 2.7	105.2 ± 7.3	110.6 ± 8.5	88.7 ± 4.6
**LC3 I**	99.0 ± 5.5	102.0 ± 5.2	104.0 ± 4.7	100.8 ± 4.3	110.5 ± 6.7	101.0 ± 5.4
**LC3 II**	104.1 ± 2.2	107.0 ± 4.7	98.1 ± 2.9	101.3 ± 2.3	113.5 ± 5.1	105.4 ± 4.7

Changes in autophagic markers Beclin-1, p62, LC3 I, LC3 II and in the activation of mTOR and p70S6K in hippocampus of 3-months old mice treated either with one (↑) or two (↑↑) or three (↑↑↑) i.p. injections of 0.9% NaCl per 24h. Semi-quantitative analysis of immunoblots was performed using Gene Tools software (Syngene, Ozyme France). The immunoreactivity of protein was normalized to β-tubulin immunoreactivity. The results are expressed as arbitrary units (%). Results are mean ± SEM for 6 mice in each group.

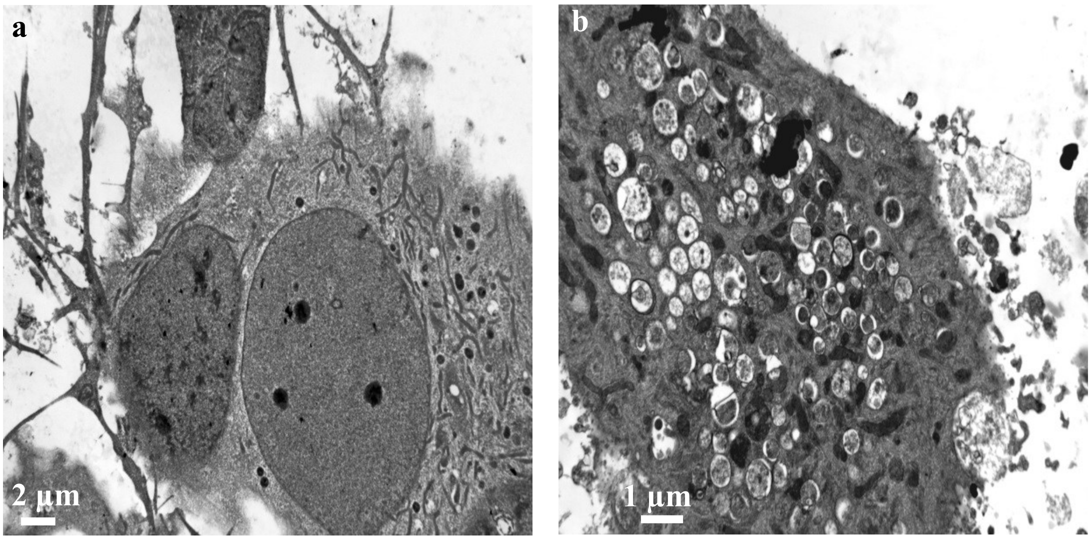

Figure 1: Accumulation of autophagic vesicles in murine primary mixed cell culture.

Murine primary mixed cell culture was prepared as described previously [50] and treated with an autophagic flux inhibitor bafilomycin A1 (50 nM) 24h before fixed with 3% glutaraldehyde in phosphate buffer saline as indicated in methods. As shown in image b, cells accumulated many autophagic vesicles in cytoplasm compared to control cell not treated with bafilomycin A1 (image a). These representative images came from 5 experiences in duplicate.
